# S100 Proteins in Fatty Liver Disease and Hepatocellular Carcinoma

**DOI:** 10.3390/ijms231911030

**Published:** 2022-09-20

**Authors:** Etienne Delangre, Ezia Oppliger, Serkan Berkcan, Monika Gjorgjieva, Marta Correia de Sousa, Michelangelo Foti

**Affiliations:** Department of Cell Physiology and Metabolism, Faculty of Medicine, University of Geneva, 1206 Geneva, Switzerland

**Keywords:** non-alcoholic fatty liver disease (NAFLD), non-alcoholic steatohepatitis (NASH), hepatocellular carcinoma (HCC), S100

## Abstract

Non-alcoholic fatty liver disease (NAFLD) is a highly prevalent and slow progressing hepatic pathology characterized by different stages of increasing severity which can ultimately give rise to the development of hepatocellular carcinoma (HCC). Besides drastic lifestyle changes, few drugs are effective to some extent alleviate NAFLD and HCC remains a poorly curable cancer. Among the deregulated molecular mechanisms promoting NAFLD and HCC, several members of the S100 proteins family appear to play an important role in the development of hepatic steatosis, non-alcoholic steatohepatitis (NASH) and HCC. Specific members of this Ca^2+^-binding protein family are indeed significantly overexpressed in either parenchymal or non-parenchymal liver cells, where they exert pleiotropic pathological functions driving NAFLD/NASH to severe stages and/or cancer development. The aberrant activity of S100 specific isoforms has also been reported to drive malignancy in liver cancers. Herein, we discuss the implication of several key members of this family, e.g., S100A4, S100A6, S100A8, S100A9 and S100A11, in NAFLD and HCC, with a particular focus on their intracellular versus extracellular functions in different hepatic cell types. Their clinical relevance as non-invasive diagnostic/prognostic biomarkers for the different stages of NAFLD and HCC, or their pharmacological targeting for therapeutic purpose, is further debated.

## 1. Introduction

**Figure 1:**
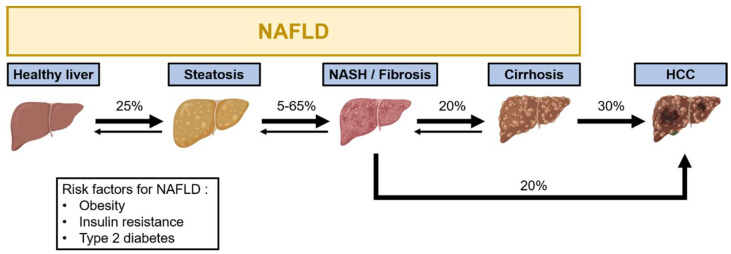
Progression from non-alcoholic fatty liver disease (NAFLD) to hepatocellular carcinoma (HCC). The sequential progression of the disease through the different stages is illustrated. The directions of arrows indicate whether stages are reversible or not. Percentages are related to the fraction of patients progressing to the next stage [8,9,10,12,13,14,15]. Associated risk factors are indicated. Artwork used to construct this figure is freely available from BioRender (https://biorender.com/ (accessed on 1 June 2022)).

Non-alcoholic fatty liver disease (NAFLD) covers a broad spectrum of hepatic disorders ranging from simple steatosis to inflammation (non-alcoholic steatohepatitis, NASH), fibrosis and cirrhosis. Eventually, hepatocellular carcinoma (HCC) can occur as an end-stage complication of the disease [1,2] (Figure 1). The progression usually spans years and may remain asymptomatic until the severe stages. Early detection and diagnosis of the disease is thus difficult, which reduces the chances to take action in time before it reaches severe and irreversible stages. Diagnostic criteria mostly rely on increased plasmatic liver enzymes, hepatomegaly and ultrasound imaging of steatosis. Infection with the hepatitis C virus (HCV), excessive alcohol consumption or long-term use of steatogenic drugs are excluding criteria for NAFLD diagnosis [3]. The main risk factors behind NAFLD development are related to metabolic disorders commonly associated with high caloric diet intake (e.g., diets excessively rich in sugar and lipids) and sedentary lifestyle, which usually lead to overweightness/obesity and associated comorbidities such as insulin resistance (IR) and type 2 diabetes [4,5]. In this regard, 70–80% of obese and type 2 diabetic patients present with NAFLD [6,7]. The worldwide prevalence of NAFLD is estimated at approximately 25% and is constantly rising [8,9], making this condition a major health burden in our society. In certain areas of the United States of America, NAFLD prevalence can even reach 50% [10]. Currently, the most effective treatment for NAFLD is a drastic change in lifestyle, including the adoption of more healthy diets and increased physical activity. In some cases, drugs increasing insulin sensitivity such as metformin may also be used [11].

*From steatosis to cirrhosis*—The first stage of NAFLD is characterized by simple steatosis. Clinically, this condition is defined as a persistent accumulation of lipid droplets in more than 5% of hepatocytes, or as intrahepatic lipids being responsible for more than 5% of the total liver weight [16]. Generally, steatosis arises when one or more of the four major pathways involved in lipid metabolism in the liver is deregulated (hepatic lipid uptake, *de novo* lipogenesis, fatty acids oxidation and the lipid export pathway) [17,18]. Insulin resistance (IR), a prevalent condition in patients suffering from obesity, also contributes to NAFLD by promoting steatosis, and as an aggravating factor leading to more severe stages of the disease. Indeed, hepatic IR increases glycogenolysis and gluconeogenesis while it decreases glycogen synthesis, thus resulting in elevated circulating glucose levels. However, in opposition to the anabolic effects of insulin, hepatic IR paradoxically promotes lipid synthesis through a still poorly elucidated mechanism [19]. IR is balanced by over-secretion of insulin by pancreatic β-cells in order to maintain euglycemia [20], resulting in hyperinsulinemia as observed in patients with NAFLD [21]. This hyperinsulinemia is considered one of the main factors enhancing hepatic lipid accumulation. Ectopic accumulation of intracellular fatty acids (FAs) also leads to increased synthesis of toxic lipid species such as ceramides [19] that further aggravate IR and lipotoxicity. Finally, systemic low-grade inflammation associated with adipose tissue hypertrophy in obese subjects and intrahepatic inflammation caused by lipotoxicity in hepatocytes also promote IR further, supporting NAFLD progression in a vicious circle [22].

Chronic liver inflammation develops after multiple hepatocyte damages caused by steatosis-associated lipotoxicity or as a consequence of low-grade systemic inflammation observed in obesity and type 2 diabetes [22]. Excessive fat accumulation in hepatocytes eventually leads to their apoptosis and damage-associated molecular pattern (DAMPs) production inducing the recruitment and activation of inflammatory cells such as resident (Kupffer cells) or non-resident macrophages, natural killer (NK) cells or T-cells in the liver [23]. The production of inflammatory mediators by hepatic immune cells in addition to obesity-related systemic low-grade inflammation signals the onset of non-alcoholic steatohepatitis (NASH) [17,24], which affect around 5–65% of patients having steatosis, depending on the population analyzed and clinical indications [8,10]. Persistent NASH then provides a favorable ground for the induction of fibrosis and formation of scare tissue in the liver.

Fibrosis is a pathophysiological response evoked by chronic inflammation and cell damage in the liver. This wound healing process is characterized by the hyperactivation of synthesis of extracellular matrix (ECM) in order to replace damaged tissue. This partially reversible mechanism arises in a disseminated way around the portal area of hepatic lobules and is mainly due to hepatic stellate cells (HSCs) activation [25]. After being activated by inflammatory factors (e.g., cytokines, transforming growth factor β (TGF-β) [26]), these cells produce excessive collagen fibers thus increasing liver stiffness and impairing the hepatic structure and function [22,27,28]. A progression to cirrhosis occurs in around 20% of the cases with the worsening of inflammation and fibrosis [12]. Cirrhosis is characterized by an excessive fibrosis extending from hepatic portal spaces to the centrolobular veins leading to a loss of hepatic lobular organization and to an exacerbated stiffness of the liver. Consequently, blood circulation is impaired, causing portal hypertension and regenerative nodules of poorly differentiated hepatocytes appear [29]. Regenerative nodules of poorly differentiated hepatocytes in cirrhotic livers are associated with a high mutation rate, which significantly increases the risk of developing HCC. The percentage of cirrhotic patients developing HCC is around 30% [13,15]. However, HCC can also arise from chronic inflammation and fibrosis in the absence of cirrhosis, as observed in approximately 20% of NAFLD patients [14].

*Hepatocellular carcinoma (HCC)*—HCC represents the main cause of primary liver cancer, which ranks in sixth position in terms of cancer incidence and in fourth position for cancer-related deaths worldwide [1,30]. The main etiological risk factors for HCC include chronic HBV and HCV infections, alcohol consumption and toxic compounds such as aflatoxins and aristolochic acid. However, in developed countries, NAFLD has become a leading risk factor for HCC [1,30]. Diagnosis of HCC usually relies on the presence in the blood of the α-fetoprotein biomarker [31], the presence of nodules detected by various imaging techniques (echography, CT-scan and MRI) [32] and the histopathological analyses of hepatic biopsies [33]. Treatment options depend on the tumor profile, the patient’s overall condition and access to medical resources. Early-stage HCC treatment includes surgical resection or liver transplantation. For intermediate-stage cancer, a catheter-based locoregional treatment can be considered and for advanced-stage HCC, treatment relies on radiotherapy and/or systemic multikinase inhibitor treatment (for example, with Sorafenib or Lenvatinib). Still, HCC treatments have low success rates and relapses are frequent [1,34].

*The S100 protein family*—Numerous genetic, epigenetic and molecular alterations have been described that contribute to NAFLD and HCC development and modulate clinical outcomes. Recently, interest has grown in investigating non-genomic alterations promoting NAFLD/HCC. Among these mechanisms, dysregulation of the expression and/or activity of members of the S100 protein family have gained increased interest as important drivers of inflammatory diseases and cancers, including NAFLD and HCC. S100 proteins are calcium-binding proteins previously associated with numerous diseases and pathological mechanisms such as inflammation and carcinogenesis [35,36,37]. Some S100 members (e.g., S100A4, S100A8/A9, S100A12 and S100B) have also been suggested to represent potential biomarkers of NAFLD-associated disorders such as obesity, type 2 diabetes, IR and inflammation [36,38]. The implication of specific S100 proteins in several types of cancer has been further documented, notably in lung and pancreatic cancers [39,40,41,42] but also in liver cancer [43,44,45]. S100 proteins are also differentially expressed in drug-resistant tumors and are therefore thought to play a role in cancer drug resistance [46]. Finally, and of major interest, S100 proteins can also be secreted and detected in body fluids with a high degree of correlation between their circulating levels and severity of particular disease. These characteristics suggest a high potential for S100 proteins as biomarkers in various pathological conditions, e.g., as suggested in melanoma malignancy prognosis [47] and/or as therapeutic targets in oncology.

In this review, we will discuss our current understanding of the contribution of S100 proteins in NAFLD development and its progression towards HCC. The intra- and extra-cellular pathophysiological functions of relevant S100 proteins in hepatic cells will be reviewed, as well as the clinical relevance of these proteins as diagnostic/prognostic biomarkers and potential therapeutic targets for NAFLD and HCC.

## 2. Structure, Expression and Regulation of S100 Proteins

### 2.1. Protein Structure

In humans, the S100 family consists of 25 members having a molecular mass between 9 and 13 kDa and encoded by 25 different genes clustered in chromosome 1 except for S100B (chromosome 21), S100G (chromosome X), S100P (chromosome 4) and S100Z (chromosome 5) [36,48]. Usually, S100 proteins form homodimers [49] with some exceptions such as S100G, which is only found as a monomer [50], or as S100A4, which can also form multimers [51] (see Table 1). Other S100 specific members can also assembly in heterodimers such as S100A1/S100B [50] and S100A8/A9 [52], which at least in the case of S100A8/A9 heterodimer is required to preserve the stability of the proteins [53]. S100 proteins are highly homologous in their sequences and structures. They contain four alpha helices and two calcium-binding EF-hand motifs connected by a flexible hinge or linker region [49]. The 14 amino acids N-terminus of the EF-hand motifs are specific to S100 proteins and characterized by a low calcium affinity. In contrast, the 12 amino acids of the C-terminus of the EF-hand motifs have a high affinity for calcium. Activation of S100 proteins is usually mediated by calcium binding to the C-terminus region, which induces a conformational change and binding to other specific cellular factors, thus triggering downstream effects in the cell. However, other activation mechanisms may exist [54,55], as illustrated by S100A10, which, in contrast to other S100 members, is constitutively active even in the absence of calcium since binding of this cation to S100A10 is prevented by mutations in its EF-hand motif [56]. However, S100A10 functions can still be indirectly dependent on calcium concentrations in some cases, as exemplified by the formation of S100A10 tetramers with Annexin A2 (ANXA2), which binds calcium in order to interact with lipid membranes [55]. Some S100 proteins are also able to bind to other cations, i.e., manganese, copper or zinc, for some specific functions, e.g., in immune defense [57,58]. The most important structural characteristics of human S100 protein members are summarized in Table 1.

Redundancy in the functions of these highly homologous S100 proteins is prevented by their very specific cell type- and context-dependent pattern of expression, their sub-cellular distribution and their secretion profiles. Finally, the hinge region of S100 proteins carries a highly variable region between the different members of this family that further defines their specific interactions with other cellular factors [39,59].

### 2.2. Expression Patterns of S100 Proteins in the Liver

**Figure 2:**
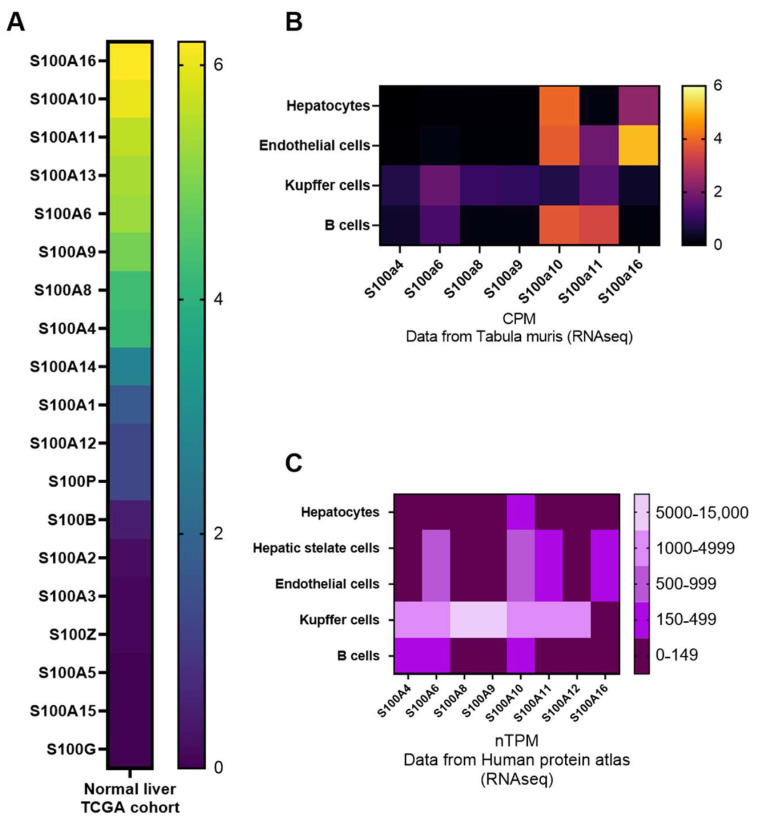
S100 mRNA expression in human liver tissues and human/mouse hepatic cells. (**A**) Relative mRNA expression (RNA seq analysis) of the different members of the S100 family in the TCGA cohort (normal human liver biopsies PMID: 25691825) acquired through the Gepia2 Cancer Database (http://gepia2.cancer-pku.cn/#index (accessed on 14 May 2022)). Values are expressed as log2(TPM + 1) (TPM—transcripts per million). (**B**) Relative mRNA expression (RNA seq analysis) of members of the S100 family (S100a4, S100a6, S100a8, S100a9, S100a10, S100a11 and S100a16) in 4 different cell types present in the livers of mice (hepatocytes, endothelial cells, Kupffer cells and B cells). Data were acquired through the tabula muris database (https://tabula-muris.ds.czbiohub.org/ (accessed on 14 May 2022)) and expression is presented as a heatmap of CPM (counts per million). (**C**) Relative mRNA expression (RNA seq analysis) of members of the S100 family (S100a4, S100a6, S100a8, S100a9, S100a10, S100a11, S100a12 and S100a16) in 5 different cell types present in the liver of humans (hepatocytes, hepatic stellate cells, endothelial cells, Kupffer cells and B cells). Data were acquired through the Human Protein Atlas database (https://www.proteinatlas.org/ (accessed on 14 May 2022)) and expression is presented as a heatmap of nTPM (transcripts per million).

S100 proteins are expressed only in vertebrates and have different cell- and tissue-specific expressions. The highest level of expression for S100 proteins is found in organs of the digestive, vascular and immune system, as well as in cancer cells [36]. In the liver specifically, mRNA expression of S100 members are low in comparison with other organs and important differences are found between specific S100 isoforms (Figure 2A), suggesting distinct functions for them in hepatic homeostasis. Single cell RNA seq analyses in humans and mice further revealed that among parenchymal (hepatocytes) and non-parenchymal cells (i.e., hepatic stellate cells, Kupffer cells, endothelial cells, fibroblasts and other immune cells), hepatocytes have the weakest expression of S100 members in basal conditions, except for S100A10, while most of the S100 are well expressed in resident Kupffer cells, thus suggesting a relevant role for them in inflammatory processes (Figure 2B,C). Of note, although S100 protein expression is restrained in normal hepatic homeostatic condition, they can strongly increase upon cellular stress, as observed following cytokines exposure [60], oxidative stress [61] or cancer [40].

### 2.3. Regulation of S100 Expression and Activity

Very few studies have investigated the molecular mechanisms in the liver, or other organs, controlling the cell-specific and/or stress-induced expression of S100 proteins, with only fragmentary information available.

Several transcription factors involved in inflammation have been described to regulate the expression of S100 members. Among them, activator protein 1 (AP-1) was reported to induce S100A10 expression [62], while Signal Transducer and Activator 3 (STAT3) had the opposite effect in the neuronal-like cell lines PC12-TrkB and N2A. The central regulator of pro-inflammatory pathways Nuclear Factor kappa B (NF-κB) was also shown to induce S100A6 expression in cardiac myocytes isolated from Sprague–Dawley rats [63], as well as S100A8 and S100A9 expression in Hep3B and Huh-7 HCC cell lines [45]. Interestingly, the S100A10 gene promoter also contains a glucocorticoid response element [55], suggesting a modulation of S100A10 expression with pathophysiological stress conditions. Supporting a drastic deregulation of S100 proteins with inflammation, in primary human gingival keratinocytes, Interleukin-1α (IL-1α) was shown to upregulate S100A8/A9 expression, while TGF-β prevented their expression [64]. TGF-β also promoted S100A11 expression in hepatic cancer cells such as Huh-7 and HepaRG [65], while growth factors also trigger S100A11 upregulation in Huh-7 [66].

Epigenetic modifications, e.g., DNA hypomethylation of CpG islands, were also reported to significantly alter the expression of S100P and S100A6 in prostate and gastric cancers, respectively, [67,68], thus underlining the importance of these S100 regulatory mechanisms in diseases. In addition, post-transcriptional regulation of S100 protein expression by microRNAs have been described. MicroRNA-dependent regulatory mechanisms play key roles in liver physiology and hepatic diseases [69]. Through in silico analyses, we identified several miRNAs predicted to target S100 members, which are involved in NAFLD and HCC (Table 2). Supporting this in silico analysis, miR-124 and miR-187 were previously reported to inhibit S100A4 expression [70,71] and miR590-5p and miR-320 were reported to decrease S100A10 expression [72,73]. Consistent with the specific and differential expression patterns of S100 proteins, our in-silico analysis could not identify single microRNAs with a general effect on most of the S100 members. In addition, S100A16 appears to be the most sensitive S100 isoform to microRNA-dependent regulation.

Finally, although the underlining regulatory mechanisms remain obscure, expression patterns depending on the cell cycle phases were observed for S100A6 in 3T3 cells [74], while seasonal- [75] and gender-dependent [75,76] serum concentrations of S100B protein were reported in humans.

## 3. General Overview of S100 Proteins Functions

S100 proteins exert pleiotropic functions in a plethora of biological processes such as calcium homeostasis, proliferation, cell migration/invasion, differentiation, apoptosis, metabolism or inflammation [36,39,46,49,77]. Each family member acts either intracellularly, mostly by binding to specific co-factors, or in an autocrine, paracrine or endocrine manner to induce physiopathological signaling following secretion in the extracellular medium. Delineating the specific contributions of the extra- and intra-cellular actions of specific S100 isoforms in biological processes remain challenging, but a better understanding of intracellular/extracellular S100 functions and signaling should provide important insights into the cell/tissue-specific role of these particular proteins.

### 3.1. Intracellular Functions

Because most S100 proteins are activated following calcium binding, it is highly probable that they might transduce raises in intracellular calcium levels into distinct cellular functions by specifically binding to a variety of intracellular co-factors [36,60,78]. However, for most of the S100 members, their impact on Ca^2+^ signaling and Ca^2+^-dependent cellular processes remains poorly characterized and needs further investigations. Currently, recent studies support a wide spectrum of essential and non-redundant cellular actions for S100 proteins [36,77], as well illustrated by the lethality of single S100 gene knockout such as S100A8 [79] or S100A16 [80] in mice.

**Figure 3:**
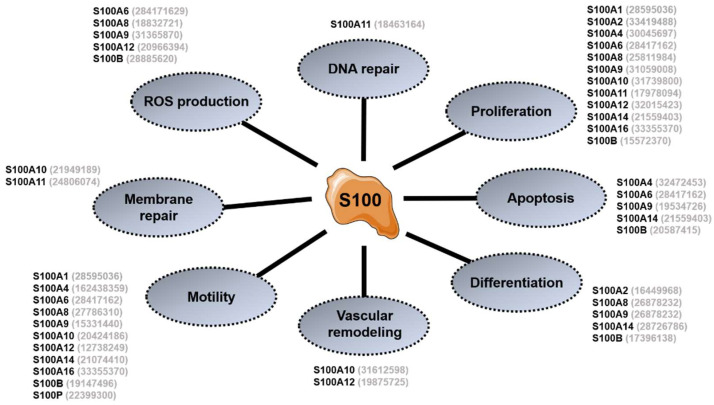
Schematic overview of the main cellular functions attributed to the activity of S100 proteins. Grey numbers in parentheses refer to the PMID of representative studies supporting the indicated function for S100 proteins.

As shown in Figure 3, S100 proteins contribute to several different key cellular functions and processes often deregulated with carcinogenesis. S100 proteins can indeed promote, or inhibit, cell proliferation and apoptosis, depending on the cell type and the cellular context, as described in the case of S100A6 [81,82] and S100A11 [83]. These dualities in the functions of specific isoforms appears to rely on complex and mechanistically still unclear bidirectional interplay of S100 isoforms with key cellular factors and cancer drivers, e.g., p53 [84], Wnt/β-catenin [85], p38 Mitogen-Activated Protein Kinases (p38 MAPK) [86], ERK [87], AKT [88], p21 [89] or NFκB [90], among others. Cell migration is also affected by numerous S100 proteins, which modulate the cytoskeleton dynamic and integrity [91,92,93,94], thereby also impacting the secretory pathways and the integrity of cell–cell junctions. Further supporting the importance of S100 specific members in cell motility and cancer metastasis, matrix metalloproteinase (MMP) expression/activity and degradation of the extra-cellular matrix (ECM) is also under the control of S100 proteins such as S100A4, S100A8, S100A9, S100A10 and S100A14 [95,96,97,98]. In addition, the intracellular production of reactive oxygen species (ROS) [99,100] and vascular remodeling [101,102] were also shown to be modulated to some extent by S100 members, e.g., S100A6, S100A10 or S100A12.

Some specific S100 proteins were also described to act as gatekeepers of the cell integrity by stimulating the repair of damaged cellular membranes or nuclear DNA. Such activities are promoted for example by S100A10 and S100A11, which form membrane repair complexes with other key factors such as ANXA2 and AHNAK [55,103], or by S100A11 interaction with RAD51 in the nucleus, which helps maintain genomic stability [104].

Finally, although secreted S100 proteins have an important role as extracellular inflammatory mediators (see below), some isoforms also contribute through their intracellular action to promote inflammation, e.g., S100A8 and S100A9, by regulating cytokine production, myeloid cell differentiation and proliferation [105]. A synthetic overview of the main S100 functions is illustrated in Figure 3.

### 3.2. Extracellular Functions

The majority of S100 proteins are secreted in body fluids, although some (e.g., S100A4, S100A13 and S100B) lack a canonical signal peptide sequence required for secretion through the classical endoplasmic reticulum/Golgi-dependent secretory pathway [106]. Specific S100 proteins could actually be released from cells following a rupture of the plasma membrane [107], allowing their passive translocation into the extracellular space. Thus, S100 proteins could act as damage-associated molecular pattern (DAMPs) able to bind specific membrane receptors on healthy cells to promote, for example, inflammatory and immune responses. S100 receptors include the Receptor for Advanced Glycation End products (RAGE) [108], the Toll-Like Receptor 4 (TLR4) [94], scavenger receptors [109] or the Fibroblast Growth Factor Receptor (FGFR) [39], which activate various intracellular signaling pathways such as NF-κB-, MAPK-, STAT3-, AP-1-, AKT-, mTOR- or Wnt/β-catenin-dependent signaling pathways (reviewed in [36]). One general outcome of S100 signaling through these various plasma membrane receptors is the production of survival proteins and growth factors, which promote proliferation [39,46]. In addition, S100 signaling triggers the secretion of other inflammatory mediators, e.g., Interleukin-1β (IL-1β) and Tumor Necrosis Factor-α (TNF-α) or chemokines, which in concert with secreted S100 proteins, induces a chemotaxis favoring the recruitment of immune cells and local inflammation. Finally, an original function in cancer was described for the extra-cellular S100A10 isoform in plasmin biogenesis through its tetramerization with ANXA2. This complex forms at the cellular surface and serves as a platform recruiting plasminogen, as well as tissue Plasminogen Activator (t-PA) and urokinase Plasminogen Activator (u-PA), two enzymes that degrade plasminogen to plasmin. Accumulation of plasmin in these conditions allows the rupture of cell–cell junctions and cell migration [55], thus promoting dissemination of tumor metastasis.

Both intra-cellular and extra-cellular functions of S100 proteins discovered to date suggest an important role of this protein family in inflammatory diseases and cancers. Discussion of our current knowledge about the role and function of S100 proteins in the liver physiology and the development of NAFLD/NASH and HCC follows.

## 4. S100 Proteins in NAFLD/NASH and HCC Development

### 4.1. Steatosis and Insulin Resistance (IR)

Deregulation of the expression and activity of several S100 proteins was suggested to significantly contribute to alterations of the lipid metabolism leading to hepatic steatosis and IR development. For example, upregulation of S100A11 [65,110] and S100A8 [111] expression was observed in the liver of NAFLD patients and various mouse models of obesity and/or steatosis, as well as in hepatocytes exposed to fatty acids, thus suggesting a strong implication of these two S100 isoforms in NAFLD development [65,110,112]. Supporting a pathological role of S100A11 upregulation in NAFLD, S100A11 overexpression in mice livers (by in vivo adenoviral transduction of hepatotropic associated-adeno viruses encoding S100A11 DNA, AAV8) fostered steatosis development [110]. On the contrary, in vivo S100A11 downregulation in hepatocytes (by in vivo adenoviral transduction of hepatotropic AAV8 encoding S100A11 specific shRNA) restrained lipid accumulation in the liver of two mouse models of diet-induced steatosis [65,110]. S100A11 was further shown to promote lipid accumulation in hepatocytes by stimulating acetylation of the forkhead box protein O1 (FOXO1) and inducing de novo lipogenesis, but not by affecting very low-density lipoprotein (VLDL) export [112].

S100A16 was also reported as a key regulator of the lipid metabolism in mice livers. Indeed, transgenic mice overexpressing constitutively S100A16 had a more severe steatosis than control mice when fed a high-fat-containing diet (HFD) inducing obesity, steatosis and IR. The inverse phenotype was observed with mice having a constitutive downregulation of S100A16 [80]. At the molecular level, S100A16 modulates AMPK activity through an interaction with calmodulin [80]. Other mechanisms might additionally be affected by S100A16 as further described in 3T3-L1 adipocytes, where S100A16 enhances lipogenesis by increasing PPARγ transcription [113,114]. In contrast to S100A11 and S100A16, S100A4 seems to protect mice against NAFLD development. Indeed, constitutive deletion of S100A4 in mice aggravated hepatic steatosis, IR and obesity development induced by HFD feeding [115]. Since in both S100A16 and S100A4 studies, gene expression for these proteins were modulated at the whole-body level in mice, the precise roles of these two isoforms in the liver specifically remains to be clearly elucidated.

**Figure 4:**
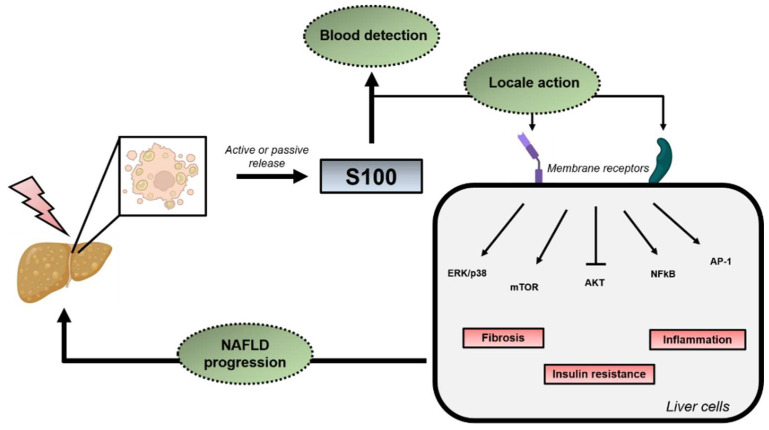
Putative model describing the impact of secreted S100 proteins in NAFLD progression. With NAFLD, damaged liver cells actively, or passively, release specific S100 proteins. Circulating S100 proteins then have the potential to stimulate TLR4/RAGE signaling, among others, in an autocrine/paracrine manner, thus further promoting IR, inflammation and/or fibrosis in a vicious circle. Of note, blood circulating S100 protein could represent relevant biomarkers of the presence and severity of NAFLD/NASH/IR. Artwork used to construct this figure is freely available from BioRender (https://biorender.com/ (accessed on 1 June 2022)).

Besides their putative intracellular roles in steatosis and/or IR development, increased serum levels of specific S100 proteins were also correlated with the presence and severity of NAFLD/IR, thus highlighting the potential of these proteins as diagnostic/predictive biomarkers. In this regard, S100A11 [110] and S100A9 [116] levels were found to be increased in blood samples of NAFLD patients, in correlation with the degree of advancement of the disease. In addition, S100A4 [117,118], S100A8/A9 heterodimers [119,120] and S100A12 [121] serum levels were also found to be increased in insulin-resistant and type 2 diabetic patients. Of note, RAGE and TLR4 signaling, which can be activated by secreted S100 proteins, are known to promote IR. Indeed, TLR4 activation by fatty acids was shown to foster IR [122] and RAGE inhibition to improve insulin sensitivity by decreasing oxidative stress [123,124]. Thus, increased extracellular levels of specific S100 isoforms have the potential to further enhance hepatic IR, thus promoting lipid accumulation in a vicious circle. Since RAGE/TLR4 receptors are also highly expressed by immune non-parenchymal liver cells (e.g., Kupffer cells, lymphocytes, neutrophils), it is likely that a similar vicious circle occurs with inflammation, thus fostering progression of simple steatosis/IR toward NASH as illustrated in Figure 4 and as described more extensively later in this review.

### 4.2. From Simple Steatosis to NASH

**Figure 5:**
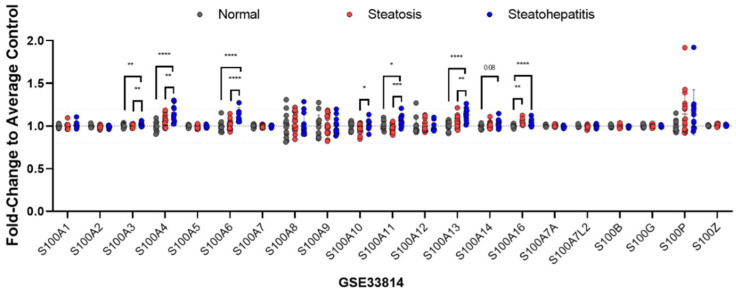
Relative mRNA expression of the different S100 family members in liver biopsies from patients diagnosed with steatosis or steatohepatitis. The relative expression is reported as fold change to control samples (mean +/− SD). Data were obtained from GSE33814 dataset using the Gene Expression Omnibus (GEO) database. One-Way ANOVA followed by Sidak’s multiple comparisons test was used for comparison between groups. * *p*-value < 0.05, ** *p*-value < 0.01, *** *p*-value < 0.001, **** *p*-value < 0.0001.

Identifying the key molecular drivers promoting the transition from hepatic benign steatosis to inflammation (NASH) and fibrosis is an important question to solve in order to design relevant therapeutic strategies. S100 proteins are theoretically good candidates to consider but their precise pathological roles and functions in the different parenchymal and non-parenchymal liver cells need to be clearly elucidated. In this regard, whether specific S100 isoforms promote or restrain liver inflammation remains unclear because of their pleiotropic effects on different liver cells. As previously mentioned, expression of S100 proteins is often upregulated by pro-inflammatory stimuli and activation of transcription factors such as AP-1, STAT3 or NFκB. Expression of several different S100 isoforms is thus often found to be increased in inflammatory diseases where they potentially exert a pro-inflammatory action [35,77,105]. Patients with NASH displayed elevated levels of plasmatic S100A8 [111], S100A9 [116] and S100A11 [65,110], while mRNA expression of several S100 isoforms, i.e., *S100A3*, *S100A4*, *S100A6*, *S100A10*, *S100A11*, *S100A13* and *S100A16*, appears to be upregulated in the hepatic tissues of patients with NASH, as assessed by in silico analyses of publicly available datasets (Figure 5). Consistent with these analyses, S100A8 [111], S100A11 [65] and S100A4 [125] overexpression was also observed in liver tissues of different mouse models of inflammation/fibrosis.

Changes in the expression of specific S100 isoforms in inflammatory cells of the liver may also deeply impact NASH onset and development through complex mechanisms. This complexity is particularly illustrated by studies examining the role of S100A8 and S100A9 in liver inflammation. S100A8 and S100A9 were, for example, reported to be highly expressed by neutrophils and macrophages (Figure 2 and [114,126]), but their expressions decrease in macrophages isolated from the livers of mice with diet-induced NASH [114]. Another study indicated that S100A8 is mostly expressed by hepatic leukocytes [100], which secrete this isoform to foster the production of pro-inflammatory cytokine TNF-α and the recruitment of other leukocytes in NASH [100]. It thus appears that leukocytes-derived S100A8 may likely promote inflammation by activating TLR4 and RAGE signaling in liver cells, as described in NK cells [115]. The role of S100A9 in liver inflammation is less clear because the constitutive gene knockout of S100A9 in a mouse model of inflammation-driven carcinogenesis did not reduce hepatic inflammation [116]. Since the deletion of S100A9 is usually accompanied by undetectable expression of the S100A8 protein [36,44,127], whether the loss of the heterodimer S100A8/A9 affected the inflammatory processes in the liver is unclear. Additional studies are thus required to clarify the intracellular role of S100A8 and S100A9 and extracellular role of the heterodimer in NASH.

Hepatic tissues and serum levels of S100A11 are also increased in NASH patients. In mice fed a choline and methionine-deficient diet (MCD), a mouse model of severe hepatic steatosis, inflammation and fibrosis, inhibition of S100A11 expression in hepatocytes was shown to restrain macrophage infiltration and expression of pro-inflammatory mediators [65]. Interestingly, secretion of S100A11 by hepatic cells might be an important driver of liver inflammation by stimulating macrophage infiltration and production of inflammatory mediators by liver cells. Indeed, studies of obese and diabetic rats fed an MCD and developing hepatic inflammation and fibrosis indicated that administration of Tranilast, a competitive inhibitor of S100A11 binding to RAGE [128], attenuated hepatic inflammation in rats [129] similarly to S100A11 downregulation in mice fed an MCD [65]. It is therefore likely that the extracellular activity of S100A11 importantly contributes to NASH development.

Finally, studies investigating the intracellular/extracellular functions of S100A4 in adipose tissue metabolism have reported contradictory results regarding dysregulation of S100A4 expression associated with obesity in different adipose tissue cells and their impacts on metabolic functions of adipose tissue and IR [38]. However, in one of these studies, the constitutive deletion of the S100A4 gene was reported to also aggravate obesity-associated hepatic inflammation in mice [115]. In addition, depletion of S100A4+ stromal cells in an alternative mouse model of NAFLD/NASH/HCC (liver-specific PTEN knockout mice) restrained hepatic inflammation but these mice also surprisingly exhibited decreased adiposity and an improved peripheral insulin sensitivity [130,131]. Evidence supports the secretion of S100A4 by inflammatory cells of the liver [132], but whether S100A4 has *per se* an important pathophysiological role in the development of hepatic inflammation, or whether the observed effect of S100A4 constitutive deletion on liver inflammation is the consequence of deep metabolic alterations in other peripheral organs such as the adipose tissues, remains to be clarified.

### 4.3. Hepatic Fibrosis

In addition to acting as inflammatory mediators, specific S100 isoforms also likely contribute to fibrogenesis development in the liver as well as in other organs. Only few S100 isoforms have been investigated in the context of fibrosis development, with S100A4 being the one better characterized in this pathological process. S100A4 was suggested to significantly contribute to fibrosis development in several different organs, including the lung [133,134], heart [135] and liver [132]. In the liver, secretion of S100A4 by inflammatory cells was shown to activate HSCs [132] leading to their transdifferentiation into myofibroblasts and production of α-Smooth Muscle Actin (α-SMA). Of note, neither HSCs, T lymphocytes nor granulocytes in the liver express S100A4, but macrophages strongly express and secrete it ([136] and Figure 2C), being therefore able to activate HSCs [132]. At the molecular level, macrophages-derived S100A4 was shown to bind to RAGE [137] and to activate the ERK pathway to promote proliferation of HSCs, a mechanism similar to the one described for S100A6 [138], a potential marker of active myofibroblasts [139]. Although the effect of S100A4 on HSCs proliferation was recently challenged [132], these data strongly suggest that macrophages recruitment at inflammation sites could promote fibrosis in part through S100A4-dependent HSCs activation. Interestingly, while hepatic tissue expression of S100A4 increases with fibrosis induced by CCl4 injection in mice, its expression decreased again during the resolution phase of fibrosis, indicating reversibility of this S100A4-dependent pro-fibrotic mechanism [140]. Finally, and of relevance, both hepatic tissue expression and serum levels of S100A4 positively correlate with the presence of fibrosis in humans [132].

S100A11 mRNA/protein expression in hepatic tissue and in the serum increase with fibrosis in both animal models and humans [65,141,142]. Of interest here, the key role of extracellular S100A11 in hepatic fibrosis is highlighted by pharmacological evidence indicating that Tranilast, an inhibitor of S100A11 binding to RAGE [128], prevents not only liver inflammation in rats fed an MCD, but also fibrosis development [129]. Finally, S100A16 is predominantly expressed in HSCs and its mRNA expression is significantly increased with NASH in humans [143]. In this regard, a recent genetic study using both S100A16 knockout and transgenic mice highlighted a hepatic pro-fibrotic role for intracellular S100A16 in HSCs [143]. Here, S100A16 expression appears to induce p53 degradation in HSCs, which in turn promotes activation of these cells via CXCR4-dependent mechanisms [143].

## 5. Implication of S100 Proteins in the Occurrence of HCC

HCC can eventually occur as a deadly end-stage of NAFLD/NASH [30]. S100 proteins have been linked to the development of many cancers [40,46], including HCC. Deregulated mRNA expression of many S100 isoforms, i.e., *S100P*, *S100A2*, *S100A5*, *S100A6*, *S100A7*, *S100A7A*, *S100A8*, *S100A9*, *S100A10*, *S100A11*, *S100A12*, *S100A13*, *S100A14*, *S100G* and *S100Z*, has indeed been associated with HCC in patients [144]. In addition, upregulation of several S100 isoforms, i.e., *S100P*, *S100A2*, *S100A6*, *S100A8*, *S100A9*, *S100A10*, *S100A11*, *S100A13* and *S100A14*, correlates with poor survival in patients [65,144]. Based on the established functions of extra/intra-cellular S100 proteins discussed above and their aberrant expression in pre-tumoral and tumoral hepatic tissues, it is therefore not surprising that these factors play key roles in carcinogenesis by either modulating the tumor microenvironment or affecting the intrinsic properties of transformed cancer cells. The expression/activity of more than half of all S100 protein members is deregulated in HCC [65] but, to date, HCC research has only focused on a few of these and there is still a lot to discover about physiological and pathophysiological roles of many S100 proteins in hepatic homeostasis and HCC development. This section summarizes the key information currently available about the four S100 members that have been investigated the most in the context of HCC development.

### 5.1. S100A4

An abnormal expression and activity of S100A4 is associated with various types of cancer [145,146,147]. In human HCC, S100A4 expression is further correlated with tumor aggressiveness and malignancy [148]. This is in accordance with several animal studies using DEN/CCl4-induced HCC, or human cancer cells xenografts, and consistently showing that S100a4 promotes cell proliferation, invasion and metastasis dissemination [149,150]. Mechanistically, extracellular S100A4 binding to RAGE induces downstream activation of proliferative pathways, e.g., β-catenin and AKT, in HCC cells [150]. On the other hand, S100A4 was shown to stimulate matrix metalloproteinase 9 (MMP-9) expression and secretion, therefore increasing cell motility and the metastatic potential of HCC cells [43,151,152,153]. Finally, while constitutive deletion of S100A4 in mice restrains the stemness, size and number of tumors induced by DEN/CCL4 administration [150], depletion of S100A4+ stromal cells in liver-specific PTEN knockout mice reduces the stem-like properties of HCC cells, but did not prevent tumorigenesis [130]. Together, these studies suggest a dual role of S100A4 on hepatic tumor initiation and progression. In contrast to the apparent preponderant role of extracellular S100A4 secreted by stromal cells in NASH, hepatic carcinogenesis indeed seems to be also affected by the intracellular expression of S100A4 in hepatocytes. Whether the pro-tumorigenic action of extracellular S100A4 relies solely on the S100A4-dependent stimulation of an inflammatory/fibrotic microenvironment or also on S100A4-mediated signaling in hepatocytes remains to be clearly established.

### 5.2. S100A8/A9

S100A9 protein expression is upregulated in human HCC [154] and high levels of *S100A8/A9* mRNAs in hepatic tumoral tissues are correlated with poor survival [144]. As previously discussed, the S100A8/A9 heterodimer is mostly expressed by immune cells and modulates inflammatory processes. Hepatocytes only weakly express these isoforms (Figure 2B,C) but immune cells producing abnormal levels of S100A8/A9 in the tumor microenvironment may deeply affect tumor initiation and progression [155]. Consistent with this concept, S100A9 is strongly expressed by tumor-associated macrophages in the liver [156]. With inflammation, NF-κB activation in hepatic cancer cells also upregulates S100A8/A9 expression, which in turn favors the formation of reactive-oxygen species (ROS) and increases cell survival [45]. Thus, in addition to generating a favorable microenvironment for hepatic tumorigenesis, aberrant expression in hepatocytes of the S100A8/A9 complex with inflammation also fosters carcinogenesis. Other reports also indicate that in vitro proliferation and invasion of hepatic cancer cells is strongly stimulated by exogenous S100A9 through activation of the MAPK/c-Jun signaling pathways [44,157,158]. This effect occurs through S100A9-dependent RAGE activation and was further confirmed in an HepG2 xenograft HCC mouse model, where injection of recombinant S100A9 stimulated cancer cells growth [157]. In vivo, the impact of S100A9 on intrinsic liver tumor development appears however more complex. Indeed, tumor cell proliferation in S100A9 genetically deficient mice was unaffected in an Mdr2^−/−^ inflammation-driven HCC mouse model [159], whereas tumor cell proliferation was decreased in a DEN-induced HCC model developing in the absence of chronic inflammation [44]. Thus, here again, the roles and functions of S100A8/A9 dimers in hepatic carcinogenesis appear complex and multiple. However, altogether intracellular and/or extracellular S100A8/A9 dimers seems to deeply impact non-parenchymal cells in the tumor microenvironment and hepatocytes to in fine exacerbate proliferation and migration/invasion features of transformed cells, thus promoting tumorigenesis and malignancy.

### 5.3. S100A10

S100A10 is significantly upregulated in human HCC and its expression negatively correlates with patient survival [160]. S100A10, by regulating the cytoskeleton dynamics and plasminogen turnover, was suggested to play an important role in cell motility. In this regard, S100A10 was described to promote invasiveness and metastasis dissemination in different types of cancers [161,162], including in in vitro transformed hepatocytes and in Hep3B mouse xenografts [160]. This oncogenic function of S100A10 was associated with its capability to form complexes with ANXA2, another potent oncogene in many cancers [55]. With hypoxia in growing tumors, upregulation of the transcription factor HIF1-α triggers ANXA2 expression and formation of stable ANXA2 complexes with S100A10 [55]. These abnormally high numbers of ANXA2/S100A10 complexes stimulate plasmin synthesis and its proteolytic activity, thus increasing cell junction rupture and ECM degradation, therefore favoring invasion and dissemination of transformed cells [163]. This increased proteolytic activity within the tumor also facilitates the recruitment of macrophages and associated inflammatory processes, thus promoting tumoral development [163].

### 5.4. S100A11

S100A11 is significantly upregulated in the liver of mouse models and humans having NASH/fibrosis [65]. With HCC, S100A11 mRNA and protein expressions further increases in mice and humans. In humans, S100A11 expression was also strongly correlated with the cancer stage, with the patient’s survival probability and secretion of this isoform by hepatic cancer cells highlighting S100A11 as a potential prognostic and diagnostic circulating biomarker for HCC [65,144]. Indeed, in other types of cancer such as melanoma, lung, ovarian and pancreatic carcinomas, S100A11 levels were found to be increased and linked with bad prognosis [39]. In vivo studies assessing the pathophysiological role of S100A11 in HCC development are currently lacking, but in vitro analyses with transformed hepatocytes cell lines provide evidence that S100A11 could exert an oncogenic activity in the liver by fostering hepatocyte proliferation [65], invasion [66] and endoplasmic reticulum stress, as well as resistance to anti-cancer drugs [65].

## 6. S100 Proteins as Potential Biomarkers and Therapeutic Targets in NAFLD/NASH and HCC

Both NAFLD/NASH and liver cancers such as HCC in humans can remain silent and poorly symptomatic until severe stages of these diseases have developed. Unfortunately, non-invasive tools for the routine detection of these diseases are cruelly lacking and the currently available pharmacological approaches to treat them are poorly efficient. In this regard, the expression, activity and secretion of specific S100 isoforms appear to be significantly deregulated at different stages of NAFLD/NASH and with HCC developing through an increased grade of severity. These features strongly suggest that S100 proteins can be used as reliable circulating or tissue biomarkers for the diagnosis and/or prognosis of these liver pathologies. Given the multiple functions of intracellular and extracellular S100 proteins in inflammation and carcinogenesis, their pharmacological targeting for therapeutic purpose also represents a promising strategy to fight these hepatic diseases.

### 6.1. S100 Proteins as Potential Biomarkers in NAFLD/NASH and HCC

**Figure 6:**
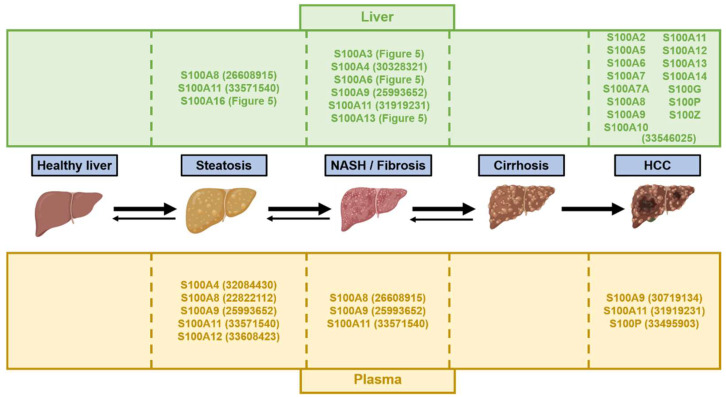
Summary of the main S100 proteins involved in the pathogenesis of NAFLD/NASH and HCC at the different stages of these diseases. The upper panel indicates the S100 proteins known to be upregulated in the liver tissue of rodents and/or humans. The lower panel indicates the main S100 proteins increased in blood samples from rodent and/or humans. The numbers in parentheses refer to the PMID of representative studies supporting the indicated function for S100 protein. Artwork used to construct this figure is freely available from BioRender (https://biorender.com/ (accession on 1 June 2022)).

The potential of specific isoforms of S100 proteins as circulating biomarkers, i.e., S100A4, S100A8, S100A9, S100A12 or S100B, has already been outlined for non-hepatic diseases such as rheumatic diseases and leukemia, respectively [35,164]. As summarized in Figure 6, the blood levels of particular S100 members are significantly increased in NAFLD/NASH and HCC and correlate with stages of increasing severity. For example, in patients suffering from obesity, increased serum levels of S100A4 were associated with liver damages and hepatic steatosis [165]. However, whether specific S100 signatures in patients’ body fluids could discriminate the different stages of NAFLD and ideally predict the risk of progression to severe stages, e.g., steatosis to NASH or NASH to cirrhosis, remains currently unclear, but future studies should provide important insights in this regard.

In HCC, numerous S100 proteins are abnormally expressed in tumoral tissues [65], but few studies investigated potential correlations between S100 protein in the blood and cancer stages (Figure 6). To date, S100A11 was shown to be highly secreted by cancer cells and HCC [65]. Serum S100A9 levels were also associated with an increased risk of recurrence and reduced overall survival in patients with HCC who underwent curative resection [166]. Finally, serum analyses of patients with HCC and patients with benign liver tumors further identified S100P blood levels as a discriminating factor [167]. Based on these pilot studies, the serological assessment of S100 proteins, in association with the detection of α-fetoprotein, an accurate marker of HCC with high specificity but poor sensitivity [168], should importantly complement the clinical arsenal of diagnostic/prognostic tools for HCC.

Microscopic examination of liver tissue biopsies remains the gold standard method for an accurate detection, staging and grading of NAFLD/NASH and HCC. Coarse analyses of hepatic tissues in humans and various animal models performed so far clearly indicate significant alterations of several S100 isoforms at different stages of NAFLD/NASH and in HCC (Figure 6 and [65]). In NAFLD/NASH, whether deregulation of specific S100 proteins expression/activity in specific hepatic cells, i.e., hepatocytes versus other non-parenchymal cells, could predict progression to severe stages such as cirrhosis, or risk of cancer development, is still a key question, but available data for example on S100A11 clearly suggest that it could be the case [65]. On the other hand, differential signatures of S100 protein expressions in HCC sample biopsies might also be indicative of bad prognosis, the presence of specific mutations or recurrence after surgery, as suggested by serum levels of S100A9 and S100P [166,167]. In-depth retrospective analyses of S100 protein expressions in clinical HCC samples should, in this respect, provide key information about their potential as diagnostic/prognostic biomarkers.

### 6.2. S100 Proteins as Therapeutic Targets for NAFLD/NASH and HCC

There are no approved therapies for NAFLD/NASH. Drastic changes in lifestyle aimed at improving dietary habits and regular physical activity, in some cases supplemented with drugs such as insulin sensitizers and lipid-lowering drugs [169], are currently the only therapeutic options to treat NAFLD/NASH [9]. NAFLD-driven HCC also remains a poorly curable disease due to its high resistance to conventional chemotherapy and radiotherapy [34]. Few patients are eligible for surgical resection and/or liver transplantation, which show some curative potential [34]. Some pharmacological compounds or antibodies are also available to treat HCC, including kinase inhibitors, e.g., sorafenib, lenvatinib and regorafenib [170,171], or monoclonal antibodies, e.g., ramucirumab, atezolizumab and bevacizumab [172,173]. However, these chemotherapies are mostly palliative and offer only an absolute survival gain of a few months for patients with significant side effects. Finally, immunotherapies have also been tested, but since HCC develop in an immunosuppressed environment, currently no single compound has proven to be effective [174]. Therefore, the discovery of new therapeutic targets to treat NAFLD/NASH and HCC remains a high medical priority. In this regard, counteracting the pathological actions of specific S100 proteins aberrantly expressed in these pathologies might be of great therapeutic interest. In particular, inhibition of the activity of extracellular S100 protein appears to represent a suitable and relevant pharmacological strategy with the advantage of not impairing vital intracellular physiological functions of these proteins.

Small pharmacological inhibitors of distinct S100 isoforms, mostly effective as anti-allergic and anti-inflammatory drugs, have been described, but their low specificity to date prevents their use in humans to treat hepatic diseases. Tranilast, for example, was reported to bind to S100A11, S100A12 and S100A13 [128,175]. Tranilast-mediated inhibition of S100A11 interaction with RAGE receptor was further shown to restrain proliferation of SW480 colon adenocarcinoma cells in vitro [128] and to decrease hepatic inflammation and fibrosis in obese and diabetic rats fed an MCD [129]. Another compound, Amlexanox, has been shown to bind S100A4, S100A12 and S100A13 [175,176] and to reduce proliferation of A431 epidermoid carcinoma cells by inhibiting S100A4 interaction with the Epidermal Growth Factor Receptor (EGFR) [176]. Olopatadine, on the other hand, was described to bind to S100A1, S100B, S100L, S100A12 and S100A13 [177] and to suppress S100A12-mediated migration of THP-1 monocytes [178]. Finally, cromolyn was also able to bind to S100A12 and S100A13 with no functional effects described as yet [175].

In addition to small pharmacological inhibitors, neutralizing antibodies of high affinity for specific S100 were developed and demonstrated to be effective against the development of specific cancers. Administration of neutralizing anti-S100A9 in a mouse model of ulcerative colitis was reported to significantly reduce inflammatory cytokine production and immune cells infiltrates, with the same antibodies also exerting a protective effect in an azoxymethane/DSS-induced colitis-associated cancer mouse model [179]. Humanized mouse chimeric antibodies against S100A8/A9 were further developed and shown to successfully inhibit melanoma mobility and lung metastasis in mice [180]. Targeting of S100A4 by a specific antibody was further demonstrated to abolish endothelial cell migration, tumor growth and angiogenesis in mouse xenografts models of M21 melanoma and MIA PaCa-2 pancreatic cancers [181]. Finally, monoclonal antibodies against S100P were reported to decrease tumor growth and metastasis in a subcutaneous and orthotopic BxPCS pancreatic tumor model [182]. Additional studies are now required to develop neutralizing antibodies against relevant circulating S100 isoforms aberrantly expressed with NAFLD/NASH and liver cancers, e.g., S100A4 and S100A11, and to investigate in vivo their therapeutic potential prior to envisaging their use in clinical settings.

## 7. Conclusions

NAFLD/NASH and HCC have a high prevalence among the global worldwide population and represent major public health concerns in our society. However, the clinical management of these diseases is hampered by the lack of relevant non-invasive diagnostic markers and effective pharmacological drugs. It is therefore of crucial importance to gain a better understanding of the molecular mechanisms responsible for the development of these diseases. In this regard, several members of the S100 protein family are highly deregulated in inflammatory diseases and cancers, including those of the liver. Although the functions of most S100 proteins are still poorly characterized, recent studies indicate that some have pleiotropic pathological functions in NAFLD/NASH fostering progression of these metabolic disorders toward severe stages and cancer development. In HCC, deregulated expression and activity of specific S100 isoforms also seems to act as key drivers of malignancy. An in-depth understanding of the pathophysiological role of intracellular and extracellular S100 proteins deregulated in liver diseases is thus likely to bring new important insights into the molecular mechanisms underlining the development and progression of these hepatic diseases. This should also allow the evaluation of the relevance of specific S100 members as new and robust biomarkers, and/or therapeutic targets, to add to the currently poor arsenal available for diagnostic/prognostic tools and chemotherapy for NAFLD/NASH and HCC, as well as for other inflammatory/fibrotic diseases and cancers.

## Figures and Tables

**Table 1:** Structural characteristics of human S100 protein members. S100 members highlighted in brown are the isoforms predominantly expressed in liver cells (see Figure 2) and described in this review as contributing to liver disease development. Data are extracted from the ExPASy database (https://www.expasy.org/resources/nextprot (accessed on 12 September 2022)). When available, PMIDs of publications describing the multimerization state of the specific S100 isoform are indicated in parenthesis. Nd: not determined.

Protein Name	Amino Acids (Molecular Weight)	Chromosomal Gene Location	EF-Hand Domain 1 (*Affinity of the Ca^2+^ Binding Site*)	EF-Hand Domain 2 (*Affinity of the Ca^2+^ Binding Site*)	Oligomerization Status
**S100A1**	94 aa (10.5 kDa)	1q21.3	EF-hand domain 1 *(low Ca^2+^ affinity)*	EF-hand domain 2 *(high Ca^2+^ affinity)*	Homodimer *(21296671)* Heterodimer with S100B *(30719832)* or S100P *(30719832)*
**S100A2**	98 aa (11.1 kDa)	1q21.3	EF-hand domain 1 *(low Ca^2+^ affinity)*	EF-hand domain 2 *(high Ca^2+^ affinity)*	Homodimer *(10951287)*
**S100A3**	101 aa (11.7 kDa)	1q21.3	EF-hand domain 1 *(low Ca^2+^ affinity)*	EF-hand domain 2 *(high Ca^2+^ affinity, **bind also Zn^2+^**)*	Homodimer and homotetramer *(18083705)*
**S100A4**	101 aa (11.7 kDa)	1q21.3	EF-hand domain 1 *(low Ca^2+^ affinity)*	EF-hand domain 2 *(high Ca^2+^ affinity)*	Homodimer and Multimeric *(19828600)*
**S100A5**	92 aa (10.7 kDa)	1q21.3	EF-hand domain 1 *(low Ca^2+^ affinity)*	EF-hand domain 2 *(high Ca^2+^ affinity)*	Homodimer *(19536568)*
**S100A6**	90 aa (10.2 kDa)	1q21.3	EF-hand domain 1 ***(Ca^2+^ affinity Nd)***	EF-hand domain 2 ***(Ca^2+^ affinity Nd)***	Homodimer *(11937060)*
**S100A7**	101 aa (11.4 kDa)	1q21.3	EF-hand domain 1 *(bind Zn^2+^)*	EF-hand domain 2 *(high Ca^2+^ affinity)*	Homodimer *(28976190)*
**S100A8**	93 aa (10.8 kDa)	1q21.3	EF-hand domain 1 *(low Ca^2+^ affinity, **bind also Zn^2+^**)*	EF-hand domain 2 *(high Ca^2+^ affinity)*	Homodimer Heterodimer or Heterotetramer with S100A9 *(17553524)*
**S100A9**	114 aa (13.2 kDa)	1q21.3	EF-hand domain 1 *(low Ca^2+^ affinity, **bind also Zn^2+^**)*	EF-hand domain 2 *(high Ca^2+^ affinity, **bind also Zn^2+^**)*	Homodimer Heterodimer or Heterotetramer with S100A8 *(17553524)*
**S100A10**	97 aa (11.2 kDa)	1q21.3	Related EF-hand domain *(**No Ca^2+^ binding**)*	Related EF-hand domain *(**No Ca^2+^ binding**)*	Heterotetramer with ANXA2 *(9886297)*
**S100A11**	105 aa (11.7 kDa)	1q21.3	EF-hand domain 1 *(low Ca^2+^ affinity)*	EF-hand domain 2 *(high Ca^2+^ affinity)*	Homodimer *(16503655)* Heterodimer with S100B *(30719832)*
**S100A12**	92 aa (10.6 kDa)	1q21.3	EF-hand domain 1 *(low Ca^2+^ affinity, **bind also Zn^2+^ and Cu^2+^**)*	EF-hand domain 2 *(high Ca^2+^ affinity, **bind also Zn^2+^ and Cu^2+^**)*	Homodimer *(18443896)* Homooligomer (tetramer or hexamer) *(19386136)*
**S100A13**	98 aa (11.5 kDa)	1q21.3	EF-hand domain 1 *(**Ca^2+^ affinity Nd**)*	**No EF-hand domain** ***(Ca^2+^ affinity Nd)***	Homodimer *(16122705)*
**S100A14**	104 aa (11.6 kDa)	1q21.3	None	EF-hand domain ***(No Ca^2+^ binding)***	Homodimer *(23197251)*
**S100A16**	103 aa (11.8 kDa)	1q21.3	Degenerated EF-hand domain 1 ***(No Ca^2+^ binding)***	EF-hand domain 2 *(high Ca^2+^ affinity)*	Homodimer *(21046186)*
**S100A7A**	101 aa (11.3 kDa)	1q21.3	EF-hand domain 1 ***(No Ca^2+^ binding, bind also Zn^2+^)***	EF-hand domain 2 *(high Ca^2+^ affinity, **bind also Zn^2+^**)*	**Nd**
**S100A7L2**	101 aa (11.3 kDa)	1q21.3	EF-hand domain 1 *(**No Ca^2+^ binding**)*	EF-hand domain 2 *(high Ca^2+^ affinity, **bind also Zn^2+^**)*	**Nd**
**S100B**	92 aa (10.7 kDa)	21q22.3	EF-hand domain 1 *(low Ca^2+^ affinity)*	EF-hand domain 2 *(high Ca^2+^ affinity)*	Homodimer *(32027773)* Heterodimer with S100A1 *(30719832)*, S100A11 *(30719832)*, S100A6 *(9925766)*
**S100G**	79 aa (9 kDa)	Xp22.2	EF-hand domain 1 *(low Ca^2+^ affinity)*	EF-hand domain 2 *(high Ca^2+^ affinity)*	Monomer *(30710283)*
**S100P**	95 aa (10.4 kDa)	4p16.1	EF-hand domain 1 *(low Ca^2+^ affinity)*	EF-hand domain 2 *(high Ca^2+^ affinity)*	Homodimer *(12808036)* Heterodimer with S100A1 *(30719832)*
**S100Z**	99 aa (11.6 kDa)	5q13.3	EF-hand domain 1 *(low Ca^2+^ affinity)*	EF-hand domain 2 *(high Ca^2+^ affinity)*	Homodimer *(11747429)*

**Table 2:** Predicted and validated microRNAs potentially regulating S100 protein expression in humans.

Protein	MicroRNA	Validated	miRTarbase ID
**S100A4**	hsa-miR-6745	no	
**S100A6**	hsa-miR-141-3p	**yes**	MIRT731072
**S100A8**	hsa-miR-125b-5p	**yes**	MIRT045918
	hsa-miR-24-3p	**yes**	MIRT052953
	hsa-miR-98-5p	**yes**	MIRT027768
**S100A9**	hsa-miR-1204	**yes**	MIRT710086
	hsa-miR-132-5p	**yes**	MIRT710087
	hsa-miR-196a-5p	**yes**	MIRT000220
	hsa-miR-4252	**yes**	MIRT4911293/MIRT710084
	hsa-miR-4679	**yes**	MIRT710085/MIRT4911292
	hsa-miR-4701-5p	**yes**	MIRT710083/MIRT4911294
	hsa-miR-588	**yes**	MIRT710082/MIRT4911295
	hsa-miR-660-3p	no	
	hsa-miR-663b	no	
	hsa-miR-766-5p	no	
**S100A10**	hsa-miR-100-5p	**yes**	MIRT048454
	hsa-miR-3122	no	
	hsa-miR-3151-5p	no	
	hsa-miR-4270	no	
	hsa-miR-4298	no	
	hsa-miR-486-3p	no	
	hsa-miR-6847-3p	no	
**S100A11**	hsa-miR-1-3p	**yes**	MIRT023889
	hsa-miR-1207-5p	**yes**	-
	hsa-miR-1293	no	
	hsa-miR-142-3p	**yes**	MIRT500051
	hsa-miR-155-5p	**yes**	MIRT020889
	hsa-miR-2861	no	
	hsa-miR-3591-5p	**yes**	MIRT500050
	hsa-miR-3609	**yes**	MIRT460529
	hsa-miR-3665	**yes**	
	hsa-miR-3934-3p	**yes**	MIRT500053
	hsa-miR-4307	**yes**	MIRT460527
	hsa-miR-4736	**yes**	-
	hsa-miR-4741	no	
	hsa-miR-548ah-5p	**yes**	MIRT460528
	hsa-miR-548az-5p	**yes**	MIRT460531
	hsa-miR-548t-5p	**yes**	MIRT460530
	hsa-miR-556-3p	**yes**	MIRT460526
	hsa-miR-6076	**yes**	MIRT500055
	hsa-miR-6134	**yes**	MIRT500056
	hsa-miR-648	**yes**	MIRT500058
	hsa-miR-6516-3p	**yes**	MIRT460525
	hsa-miR-6797-3p	**yes**	MIRT500054
	hsa-miR-7854-3p	**yes**	MIRT500057
	hsa-miR-876-3p	**yes**	MIRT500052
**S100A12**	hsa-miR-146a-5p	**yes**	MIRT437615 MIRT437621
	hsa-miR-4505	no	
	hsa-miR-4710	no	
	hsa-miR-5787	no	
	hsa-miR-6858-5p	no	
**S100A16**	hsa-miR-1-3p	**yes**	MIRT024074
	hsa-miR-1207-5p	**yes**	-
	hsa-miR-1247-3p	**yes**	-
	hsa-miR-1249-5p	**yes**	-
	hsa-miR-1293	**yes**	-
	hsa-miR-1912-3p	no	
	hsa-miR-193b-3p	**yes**	MIRT016530
	hsa-miR-24-3p	**yes**	-
	hsa-miR-2467-5p	**yes**	-
	hsa-miR-3116	no	
	hsa-miR-3184-5p	no	
	hsa-miR-363-5p	**yes**	-
	hsa-miR-3929	**yes**	-
	hsa-miR-423-5p	no	
	hsa-miR-4478	**yes**	-
	hsa-miR-4481	no	
	hsa-miR-4510	**yes**	-
	hsa-miR-4514	**yes**	-
	hsa-miR-4537	**yes**	-
	hsa-miR-4689	**yes**	-
	hsa-miR-4692	**yes**	-
	hsa-miR-4695-5p	**yes**	-
	hsa-miR-4736	**yes**	-
	hsa-miR-4746-3p	**yes**	-
	hsa-miR-4784	**yes**	-
	hsa-miR-498-5p	no	
	hsa-miR-541-3p	**yes**	-
	hsa-miR-6085	no	
	hsa-miR-6127	**yes**	-
	hsa-miR-6129	**yes**	-
	hsa-miR-6130	**yes**	-
	hsa-miR-6515-5p	no	
	hsa-miR-665	**yes**	-
	hsa-miR-6715b-5p	**yes**	-
	hsa-miR-6721-5p	**yes**	-
	hsa-miR-6745	**yes**	-
	hsa-miR-6756-5p	**yes**	-
	hsa-miR-6760-5p	no	
	hsa-miR-6766-5p	**yes**	-
	hsa-miR-6774-5p	**yes**	-
	hsa-miR-6775-3p	**yes**	-
	hsa-miR-6776-5p	**yes**	-
	hsa-miR-6791-5p	**yes**	-
	hsa-miR-6808-5p	**yes**	-
	hsa-miR-6813-5p	no	
	hsa-miR-6827-5p	**yes**	-
	hsa-miR-6847-5p	no	
	hsa-miR-6858-5p	**yes**	-
	hsa-miR-6884-5p	**yes**	-
	hsa-miR-6893-5p	**yes**	-
	hsa-miR-7150	**yes**	-
	hsa-miR-7157-5p	no	
	hsa-miR-7160-5p	**yes**	-
	hsa-miR-765	**yes**	-

## Data Availability

Not applicable.

## Abbreviations

DAMP: Damage Associated Molecular Pattern; ECM: Extracellular Matrix; FA: Fatty Acid; FFA: Free Fatty Acid; HBV: Hepatitis B Virus; HCC: Hepatocellular Carcinoma; HCV: Hepatitis C Virus; HFD: High Fat Diet; HSC: Hepatic Stellate Cell; IR: Insulin Resistance; MCD: Methionine/Choline Deficient Diet; MMP: Matrix Metalloproteinase; NAFLD: Non-Alcoholic Fatty Liver Disease; NASH: Non-Alcoholic Steatohepatitis; NK: Natural Killer; ROS: Reactive Oxygen Species; VLDL: Very Low-Density Lipoprotein
